# Mechanistic insights into the anti-neuroinflammatory effects of cassia *obtusifolia* in Parkinson’s disease: a network pharmacology-based study

**DOI:** 10.3389/fnagi.2025.1730009

**Published:** 2026-01-09

**Authors:** Xinfu Lian, Yongjun Bai, Rong Xie, Wang Du, Lingbo Ma, Yuqian Jiang

**Affiliations:** 1 First Section, Department of Neurology, Zhuhai Hospital, Guangdong Provincial Hospital of Traditional Chinese Medicine, Zhuhai, Guangdong, China; 2 Department of Neurology, Zhuhai Hospital, Guangdong Provincial Hospital of Traditional Chinese Medicine, Zhuhai, Guangdong, China; 3 Department of Brain Disease, Zhuhai Hospital, Guangdong Provincial Hospital of Traditional Chinese Medicine, Zhuhai, Guangdong, China

**Keywords:** *Cassia obtusifolia*, Parkinson’s disease, neuroinflammation, network pharmacology, NF-κB pathway

## Abstract

**Background:**

Parkinson’s disease (PD) is a chronic neurodegenerative disorder that is closely associated with neuroinflammation, yet effective anti-inflammatory therapies remain limited. This study aimed to elucidate the potential mechanisms of *Cassia obtusifolia* in mitigating PD-associated neuroinflammatory responses.

**Methods:**

Network pharmacology was employed to identify bioactive compounds, candidate targets, and enriched pathways, followed by protein–protein interaction (PPI) analysis and molecular docking. Rhein, a representative compound, was further validated in LPS-induced BV2 microglial cells using CCK-8, NO detection, ELISA, and Western blot assays.

**Results:**

A total of 114 candidate targets were identified, with enrichment highlighting NF-κB, MAPK, and NLRP3 inflammasome pathways. Molecular docking revealed strong binding affinity between rhein and NF-κB p65. *In vitro*, rhein significantly reduced the production of inflammatory mediators and suppressed p65 phosphorylation in BV2 cells.

**Conclusion:**

*Cassia obtusifolia* exerts multi-target anti-neuroinflammatory effects, supporting its potential as a therapeutic candidate for PD and providing a foundation for further translational studies.

## Introduction

Parkinson’s disease (PD) is the second most prevalent neurodegenerative disorder worldwide, primarily affecting the elderly population and posing an increasing burden on public health systems (Höglinger et al., 2024). Clinically, PD manifests as motor dysfunctions-including tremor, rigidity, and bradykinesia, as well as diverse non-motor symptoms that significantly impair quality of life (Jerčić et al., 2024; Peña-Zelayeta et al., 2025). Pathologically, PD is characterized by the progressive degeneration of dopaminergic neurons in the substantia nigra and the abnormal aggregation of α-synuclein. Accumulating evidence highlights neuroinflammation as a critical contributor to PD pathogenesis rather than a secondary consequence of neuronal loss (Kopp et al., 2022). Activated microglia, as the resident immune cells of the central nervous system, secrete pro-inflammatory cytokines and reactive oxygen species (ROS), thereby amplifying neuronal injury and accelerating neurodegeneration.

Although various pharmacological and surgical interventions have improved symptomatic control, current therapies fail to modify the disease course. Dopamine replacement therapy, the mainstay treatment, temporarily alleviates motor dysfunction but does not prevent neuronal loss or alter disease progression. Similarly, advanced interventions such as deep brain stimulation can improve motor symptoms without targeting the underlying inflammatory mechanisms (Wu et al., 2023; Wu et al., 2024). Conventional anti-inflammatory agents are limited by insufficient specificity and systemic side effects, underscoring the need for safer and more effective approaches to modulate neuroinflammation in PD.

Traditional Chinese medicine (TCM), characterized by its multi-component and multi-target mechanisms, has gained attention for its therapeutic potential in neurodegenerative diseases. *Cassia obtusifolia* L. (Jue Ming Zi), traditionally used to “clear the liver and improve eyesight,” and pharmacological studies have demonstrated its broad spectrum of biological activities (Ali et al., 2021; Ju et al., 2010). *Cassia obtusifolia* L. Contains abundant anthraquinones such as rhein, emodin, and chrysophanol, which have demonstrated anti-inflammatory, antioxidant, and neuroprotective activities (Fu et al., 2024). Notably, these compounds have been reported to regulate key signaling pathways implicated in neuroinflammation, including NF-κB and MAPK, suggesting that *Cassia obtusifolia* holds promise as a potential therapeutic candidate for PD.

However, systematic investigations into the anti-neuroinflammatory effects of *Cassia obtusifolia* in PD remain scarce. Most existing work has centered on individual compounds or single pathways, without integrating bioinformatics approaches and experimental validation to uncover the herb’s comprehensive mechanisms of action. To bridge this gap, the present study integrates network pharmacology, molecular docking, and cell-based experiments in LPS-induced BV2 microglial models. By combining computational predictions with experimental evidence, this work aims to elucidate the potential mechanisms through which *Cassia obtusifolia* mitigates neuroinflammation in PD and to provide a scientific basis for the further development of TCM in neurodegenerative disease therapy.

## Materials and methods

### Overall workflow

**FIGURE 1:**
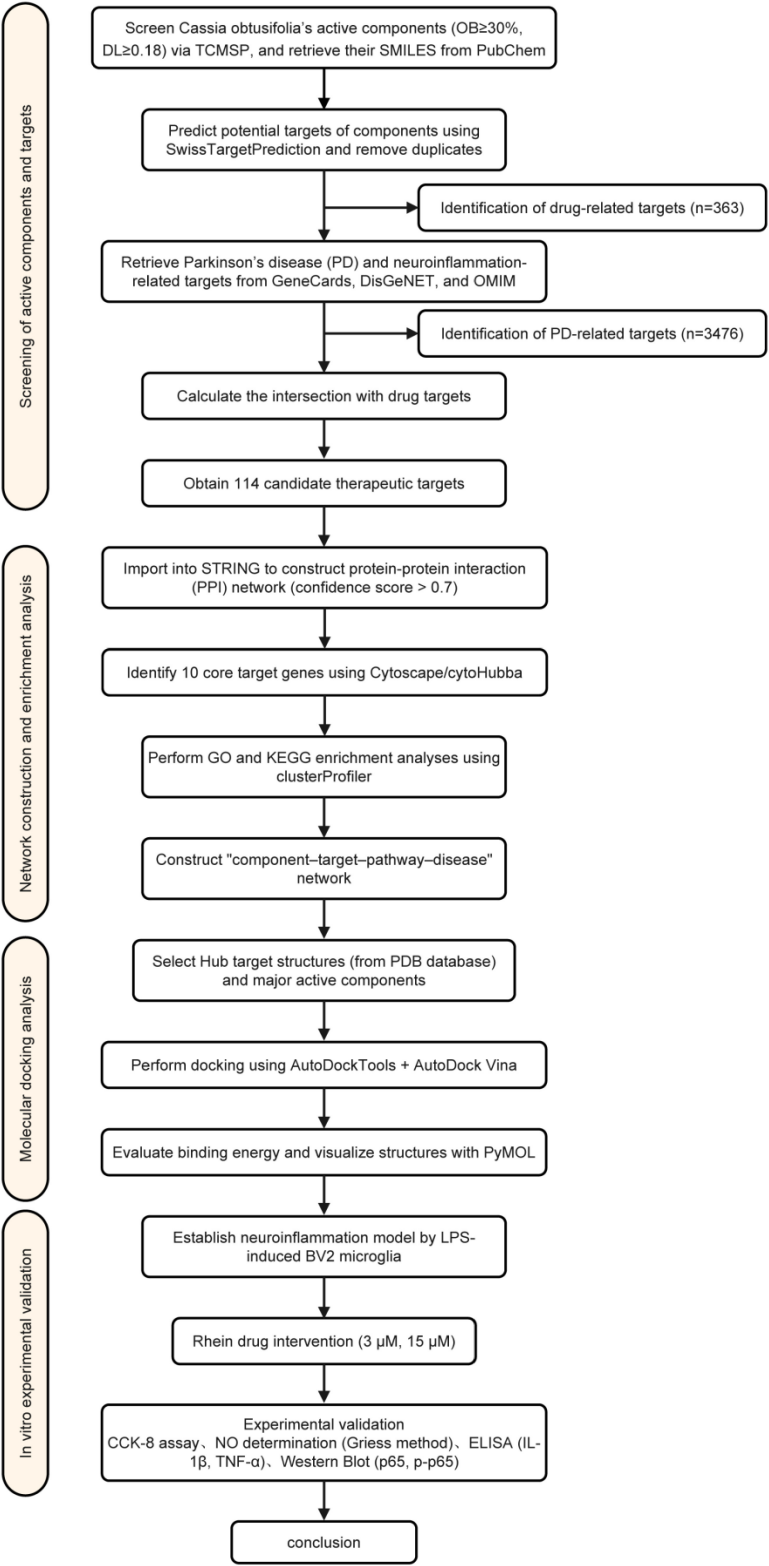
Comprehensive strategy diagram.

To provide an overview of the methodological framework, we first present a schematic diagram summarizing the entire workflow, from compound screening and target identification to molecular docking and *in vitro* validation (Figure 1). The detailed procedures for each step are described below.

### Active compound and target identification

The chemical constituents of *Cassia obtusifolia* were retrieved from the Traditional Chinese Medicine Systems Pharmacology database (TCMSP).^1^ Active compounds were screened based on oral bioavailability (OB ≥ 30%) and drug-likeness (DL ≥ 0.18) thresholds. The canonical SMILES structures of candidate compounds were obtained from PubChem.^2^ Potential protein targets were predicted using SwissTargetPrediction,^3^ with the species limited to *Homo sapiens*. Target names were standardized to gene symbols using UniProt.^4^ After integration and removal of duplicates, a compound–target dataset was generated for subsequent network construction.

### Disease target collection and intersection analysis

Parkinson’s disease-and neuroinflammation-related genes were collected by searching GeneCards,^5^ DisGeNET,^6^ and OMIM^7^ databases with the keywords “Parkinson’s disease” and “neuroinflammation.” Following the removal of duplicate entries, targets with high relevance scores were retained. The overlapping targets between the disease-associated targets and the *Cassia obtusifolia*–derived targets were identified and visualized using an online Venn diagram tool, yielding candidate therapeutic targets for further analysis.

### Functional and pathway enrichment analysis

The candidate targets were analyzed for Gene Ontology (GO) and Kyoto Encyclopedia of Genes and Genomes (KEGG) enrichment using the clusterProfiler package in R. Terms with *p* < 0.05 were considered significant. GO results were categorized into biological process (BP), cellular component (CC), and molecular function (MF) domains, and KEGG analyses summarized enriched signaling pathways. Pathways closely associated with neuroinflammation, such as NF-κB, NLRP3 inflammasome, MAPK, Toll-like receptor, TNF, and IL-17 signaling, were emphasized. Results were visualized as bubble plots to display the top enriched terms.

### Protein–protein interaction network and hub target identification

The candidate targets were queried in STRING database^8^ to construct a protein–protein interaction (PPI) network, with the organism specified as *Homo sapiens* and a minimum interaction threshold of 0.7. The PPI network was visualized using Cytoscape.^9^ Topological parameters were analyzed with the NetworkAnalyzer plugin, and hub targets were identified with the cytoHubba plugin based on degree centrality.

### Molecular docking

The 3D structures of hub proteins were downloaded from the RCSB Protein Data Bank (PDB),^10^ applying a resolution cutoff of 2.6 Å. Active compounds, including rhein, emodin, and chrysophanol, were retrieved from PubChem and structurally optimized prior to docking. Protein and ligand structures were prepared with AutoDockTools and converted to PDBQT format. Docking simulations were performed using AutoDock Vina,^11^ and binding affinities were reported as binding energy (kcal/mol). The docking conformations were visualized using PyMOL,^12^ and a heatmap summarizing docking scores was generated to compare binding capacities among compound–target pairs.

### Cell model and grouping

Murine BV2 microglial cells were obtained from the Cell Bank of the Chinese Academy of Sciences (Shanghai, China; Cat. No. SCSP-M5208). This cell line has been identified by the supplier and is cryopreserved in a medium containing 90% fetal bovine serum and 10% dimethyl sulfoxide. After thawing and passage, it is used for all experiments. The cells are cultured in DMEM medium (Thermo Fisher Scientific, United States; item number 11965092), supplemented with 10% fetal bovine serum (Thermo Fisher Scientific, United States; item number A5670701), and maintained in a humidified incubator at 37°C and 5% CO2. All cell culture operations follow the guidelines of the International Organization for Standards in *In Vitro* Research (OECD), and are conducted under aseptic conditions using a biosafety cabinet (Thermo Fisher Scientific, United States; 1500 series B2 type) to ensure aseptic operation and personnel safety. Neuroinflammation was induced by stimulation with lipopolysaccharide (LPS, Sigma-Aldrich, United States; Cat. No. L2630) at a final concentration of 100 ng/mL for 24 h, which is a well-established method for mimicking microglial activation (Yan et al., 2018). Rhein (≥ 98% purity, Aladdin, Shanghai, China; Cat. No. R111265, CAS: 478–43–3) was dissolved in DMSO (Thermo Fisher Scientific, United States; Cat. No. 20688) and applied 1 h prior to LPS stimulation (Zheng et al., 2020). The final concentration of DMSO was kept below 0.1% to minimize solvent-related cytotoxicity. The cells were divided into five groups: Control (untreated), LPS, Vehicle (LPS + DMSO), LPS + Rhein_L (3 μM), and LPS + Rhein_H (15 μM).

### Cell viability assay

Cell viability was assessed using the Cell Counting Kit-8 (CCK-8, Dojindo Laboratories, Japan; Cat. No. CK04). BV2 cells were seeded into 96-well plates and treated according to the experimental grouping scheme. Following 2 h incubation with the CCK-8 reagent, the absorbance at 450 nm was measured using a microplate reader (BioTek, United States). Each treatment group was assayed in triplicate (*n* = 3) within each independent experiment, and a total of three independent experiments (*N* = 3) were performed. The obtained data were normalized to those of the control group.

### Nitric oxide measurement

Nitric oxide levels in culture supernatants were determined using a commercial NO assay kit (Beyotime Biotechnology, Shanghai, China; Cat. No. S0021S). Nitrite, an index of NO generation, was quantified by the Griess reaction according to the manufacturer’s protocol. Absorbance was measured at 540 nm on a microplate reader (BioTek, United States), and NO concentrations were calculated from a sodium nitrite standard curve. Each experimental group was measured in triplicate (*n* = 3) per independent experiment, and the experiment was repeated three times independently (*N* = 3).

### Enzyme-linked immunosorbent assay

The concentrations of interleukin-1β (IL-1β) and tumor necrosis factor-α (TNF-α) in the culture supernatant were quantified using ELISA kits (eBioscience, Thermo Fisher Scientific, United States; IL-1β Cat. No. BMS6002, TNF-α Cat. No. BMS607-3). The supernatant was collected following the respective treatments and processed in strict accordance with the manufacturer’s protocols. Absorbance was measured at 450 nm on a microplate reader (BioTek, United States), and cytokine concentrations were calculated from standard curves. Each experimental group was measured in triplicate (*n* = 3) per independent experiment, and the experiment was repeated three times independently (*N* = 3).

### Western blotting

Cells were lysed in RIPA buffer supplemented with protease and phosphatase inhibitors (Beyotime, China; Cat. No. P0013B). Equal amounts of protein were resolved by SDS-PAGE and transferred to PVDF membranes (Millipore, United States; Cat. No. IPVH00010). After blocking with 5% BSA (Solarbio, Beijing, China, Cat. No. SW3015), the membranes were incubated overnight at 4°C with primary antibodies against p65 (Abcam, United Kingdom; Cat. No. ab32536), phospho-p65 (Abcam, United Kingdom; Cat. No. ab76302), and β-actin (Abcam, United Kingdom; Cat. No. ab8227). Following incubation with HRP-conjugated secondary antibodies (Abcam, United Kingdom; Cat. No. ab6721), the protein bands were visualized with an ECL kit (Thermo Fisher Scientific, United States; Cat. No. 32106) and quantified with ImageJ software (NIH, United States). Protein samples for each treatment group were collected from three independent cell cultures (*N* = 3). A representative blot from one experiment is shown, and quantitative data are presented as the mean of three independent experiments.

### Statistical analysis

All bioinformatics analyses, including target enrichment and visualization, were performed in R software (version 4.3.2). For *in vitro* experiments, data were analyzed using IBM SPSS Statistics 23.0 (IBM Corp., Armonk, NY, United States). Results were expressed as mean ± standard deviation (SD) from at least three independent experiments. Group comparisons were conducted using one-way ANOVA, followed by Tukey’s *post hoc* test when variances were homogeneous, or Dunnett’s T3 test when variances were unequal. A two-tailed *p* < 0.05 was considered statistically significant.

## Results

### Candidate target identification of *Cassia obtusifolia* against Parkinson’s disease

**FIGURE 2:**
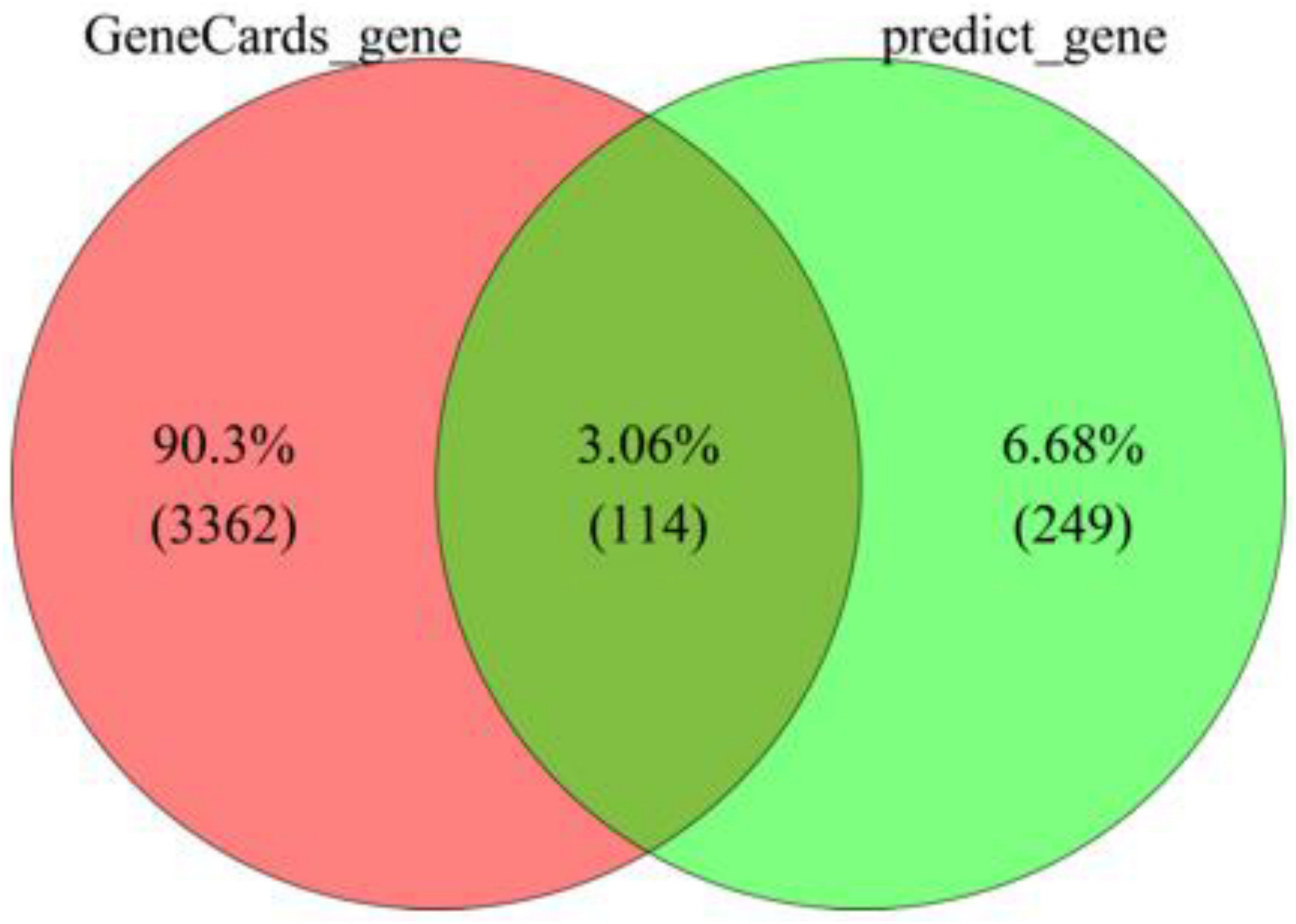
Venn diagram of predicted drug-related targets and PD-associated genes.

Using the TCMSP and PubChem databases, a total of 14 active compounds from *Cassia obtusifolia* were identified, among which 11 had available SMILES structures. Through SEA and SwissTarget Prediction analyses, 363 potential drug-related targets were obtained after removing duplicates. Meanwhile, 3476 PD-related genes were retrieved from the GeneCards database (Relevance score > 10). By intersecting the drug-related targets with PD-associated genes, 114 common targets were identified as candidate therapeutic targets (Figure 2).

### PPI Network construction and hub gene identification

**FIGURE 3:**
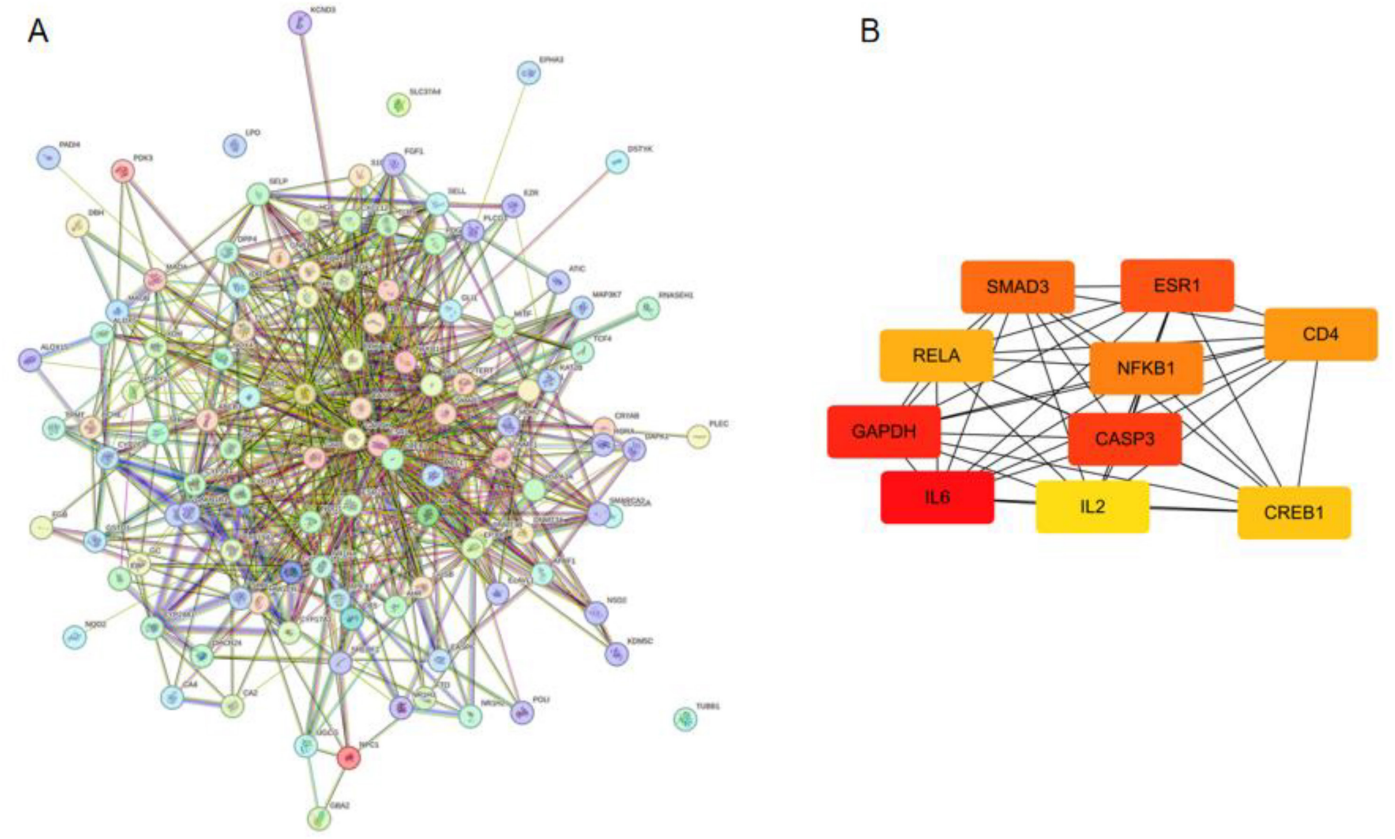
Protein–protein interaction analysis of candidate targets. **(A)** PPI network of 114 intersecting genes constructed using the STRING database. **(B)** The top 10 hub genes identified by degree centrality in Cytoscape.

The 114 candidate targets were imported into the STRING database to construct a PPI network. After applying a confidence score threshold of > 0.7, a highly interconnected network was obtained (Figure 3A). Topological analysis using the cytoHubba plugin in Cytoscape identified 10 hub genes based on degree centrality, namely IL6, GAPDH, CASP3, ESR1, SMAD3, NFKB1, CD4, RELA, CREB1, and IL2 (Figure 3B).

### GO and KEGG enrichment analysis

**FIGURE 4:**
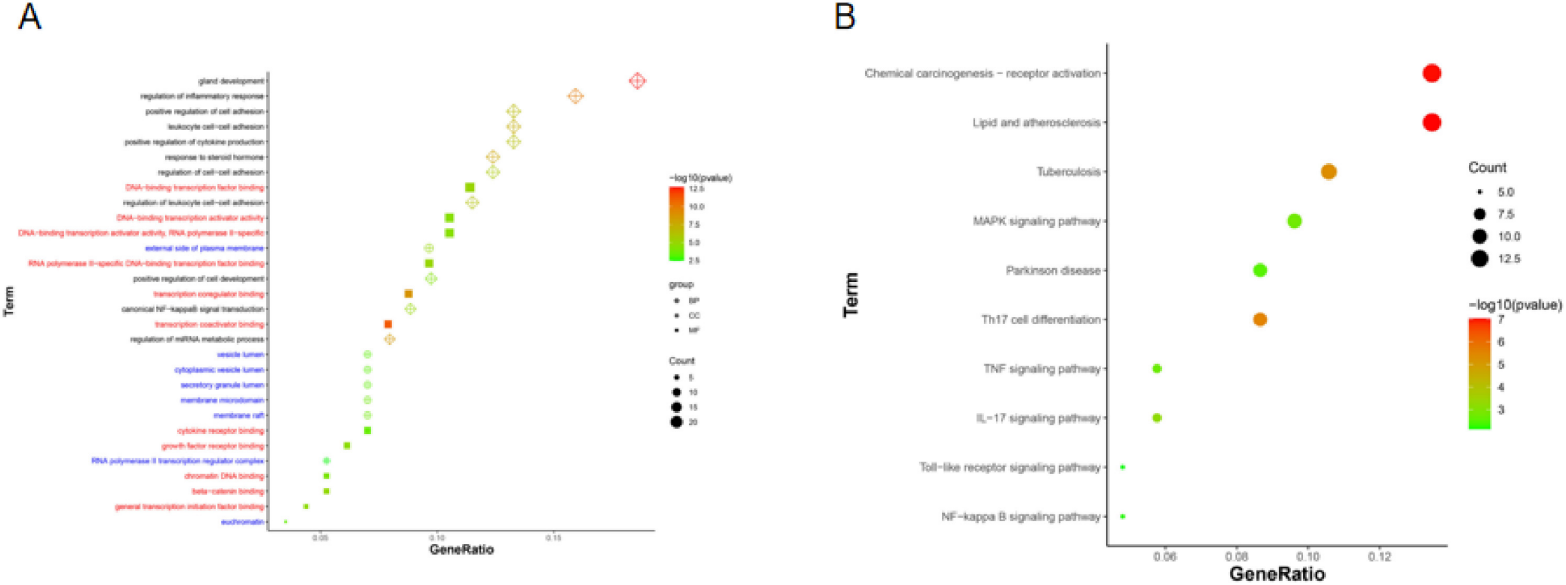
Functional enrichment analysis of candidate targets. **(A)** GO enrichment results across biological process (BP), cellular component (CC), and molecular function (MF) categories. The x-axis indicates the gene ratio, bubble size represents the number of enriched genes, and color corresponds to the adjusted *p*-value. **(B)** KEGG pathway enrichment results. The x-axis indicates the gene ratio, bubble size represents the number of enriched genes, and color corresponds to the adjusted *p*-value.

To further explore the biological functions of the 114 candidate targets, GO and KEGG enrichment analyses were performed. GO analysis revealed significant enrichment in multiple biological processes, molecular functions, and cellular components, including regulation of inflammatory response, leukocyte cell-cell adhesion, cytokine receptor binding, and transcription factor activity (Figure 4A). KEGG pathway analysis demonstrated that the candidate targets were primarily enriched in inflammation-related signaling pathways, including MAPK, TNF, IL-17, Toll-like receptor, NF-κB pathways, as well as pathways associated with Parkinson’s disease (Figure 4B).

### Construction of the compound–target–pathway–disease network

**FIGURE 5:**
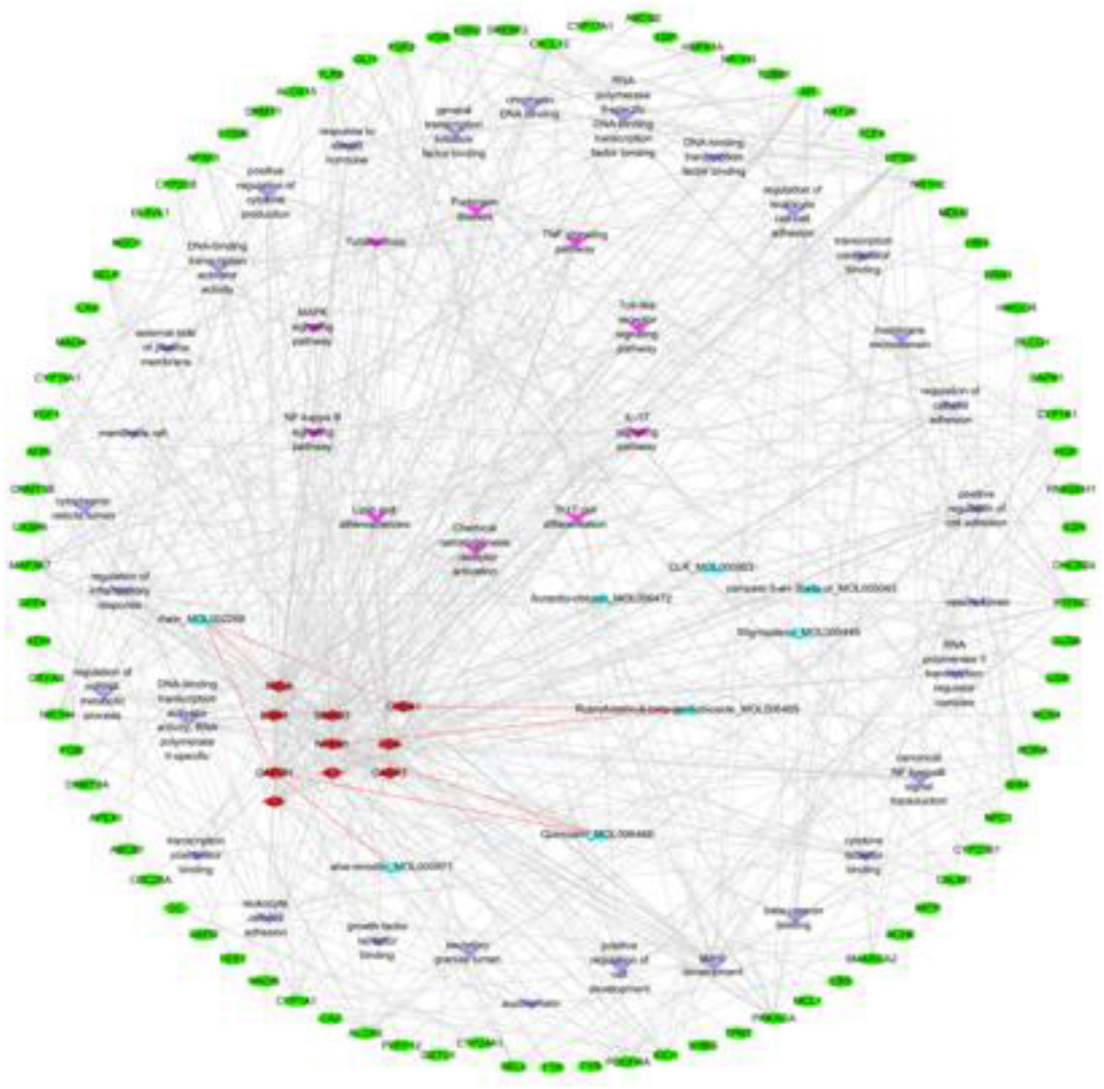
Network of compound–target–pathway–disease interactions. Purple V-shaped nodes represent KEGG pathways, light purple V-shaped nodes represent GO terms, green ellipses represent non-hub genes, red diamonds represent hub genes, and blue triangles represent active compounds. Red connecting lines indicate stable compound–target interactions.

To elucidate the interaction relationships, we integrated the top 30 GO terms and 10 KEGG pathways with PPI data from STRING (confidence score > 0.75). Eight active compounds from *Cassia obtusifolia* were mapped to 10 hub genes, and their interactions with enriched pathways were systematically combined to construct a compound–target–pathway–disease network (Figure 5). The network visually demonstrates the complex interplay among active compounds, hub targets, and enriched pathways.

### Molecular docking analysis

**FIGURE 6:**
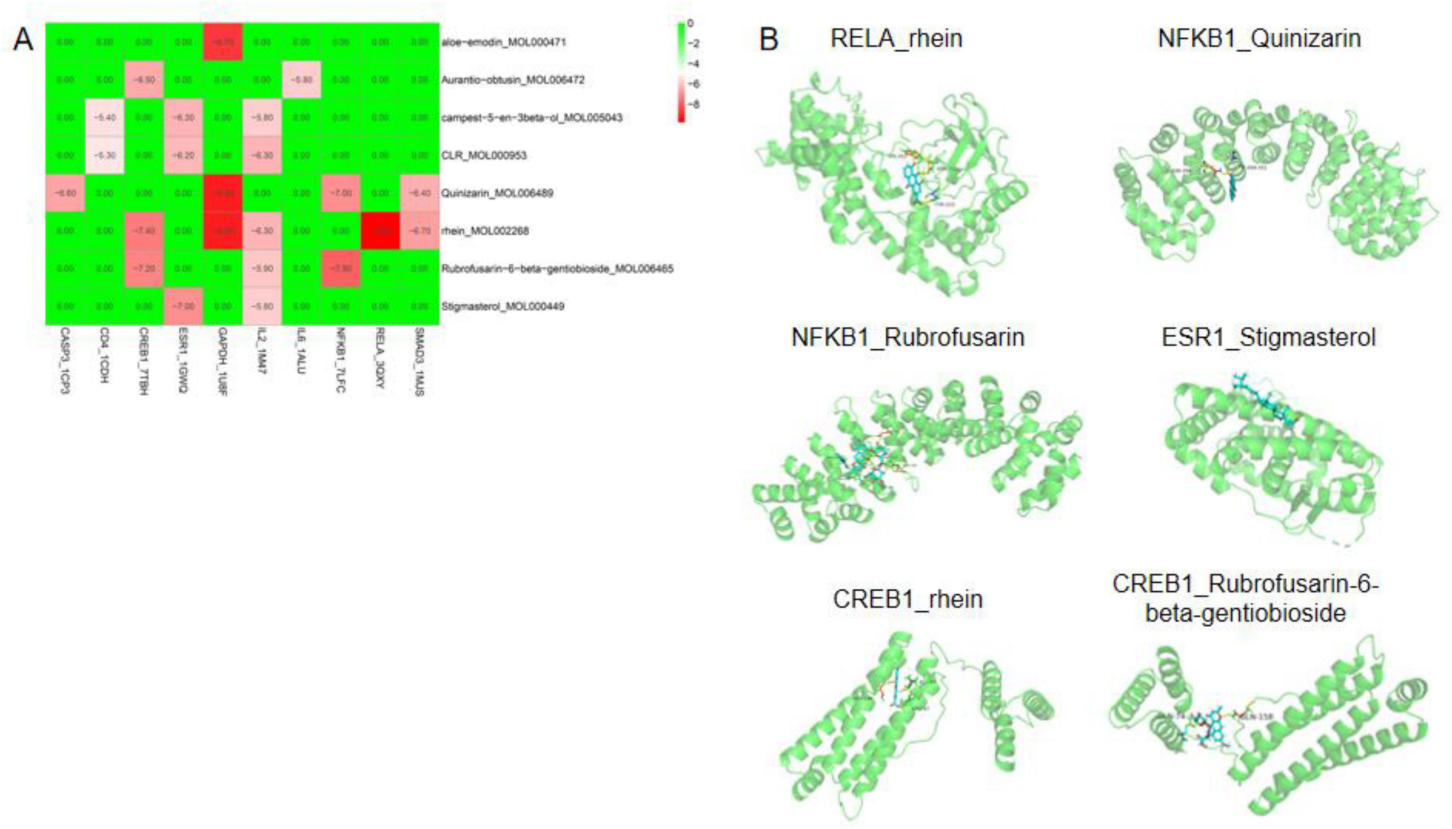
Molecular docking analysis of *Cassia obtusifolia* compounds with hub proteins. **(A)** Heatmap of docking scores for eight active compounds and ten hub proteins. The color scale represents binding affinity (kcal/mol), with red indicating stronger predicted binding and green indicating weaker binding. **(B)** Representative docking conformations of six compound–target complexes.

Molecular docking was performed for 23 compound–target pairs using AutoDock Vina to evaluate binding interactions. Binding energies were calculated, and nine complexes with binding energy ≤ -7.0 kcal/mol were identified. The docking heatmap illustrated the energy distribution across all pairs (Figure 6A). Binding energies ranged from -9.8 to -7.0 kcal/mol, with RELA–rhein complex showing the lowest value (-9.8 kcal/mol). The quantitative analysis of the conformational structure revealed the key hydrogen bond interactions that underlie these strong binding affinities (Table 1). Other notable pairs included NFKB1–rubrofusarin (-7.9 kcal/mol), CREB1–rhein (-7.4 kcal/mol), CREB1–rubrofusarin-6-beta-gentiobioside (-7.2 kcal/mol), ESR1–stigmasterol (-7.0 kcal/mol), and NFKB1–quinizarin (-7.0 kcal/mol). Representative docking conformations of these six compound–target complexes are shown in Figure 6B and Table 1.

**TABLE 1:** Summary of the latest quantitative parameters of representative complexes.

Protein	Compound	Binding energy (kcal/mol)	No. of H-bonds
RELA	Rhein	−9.8	3
NFKB1	Rubrofusarin	−7.9	7
CREB1	Rhein	−7.4	3
CREB1	Rubrofusarin-6-beta-gentiobioside	−7.2	2
ESR1	Stigmasterol	−7.0	0
NFKB1	Quinizarin	−7.0	2

### *In vitro* validation of rhein effects in LPS-stimulated BV2 microglia

**FIGURE 7:**
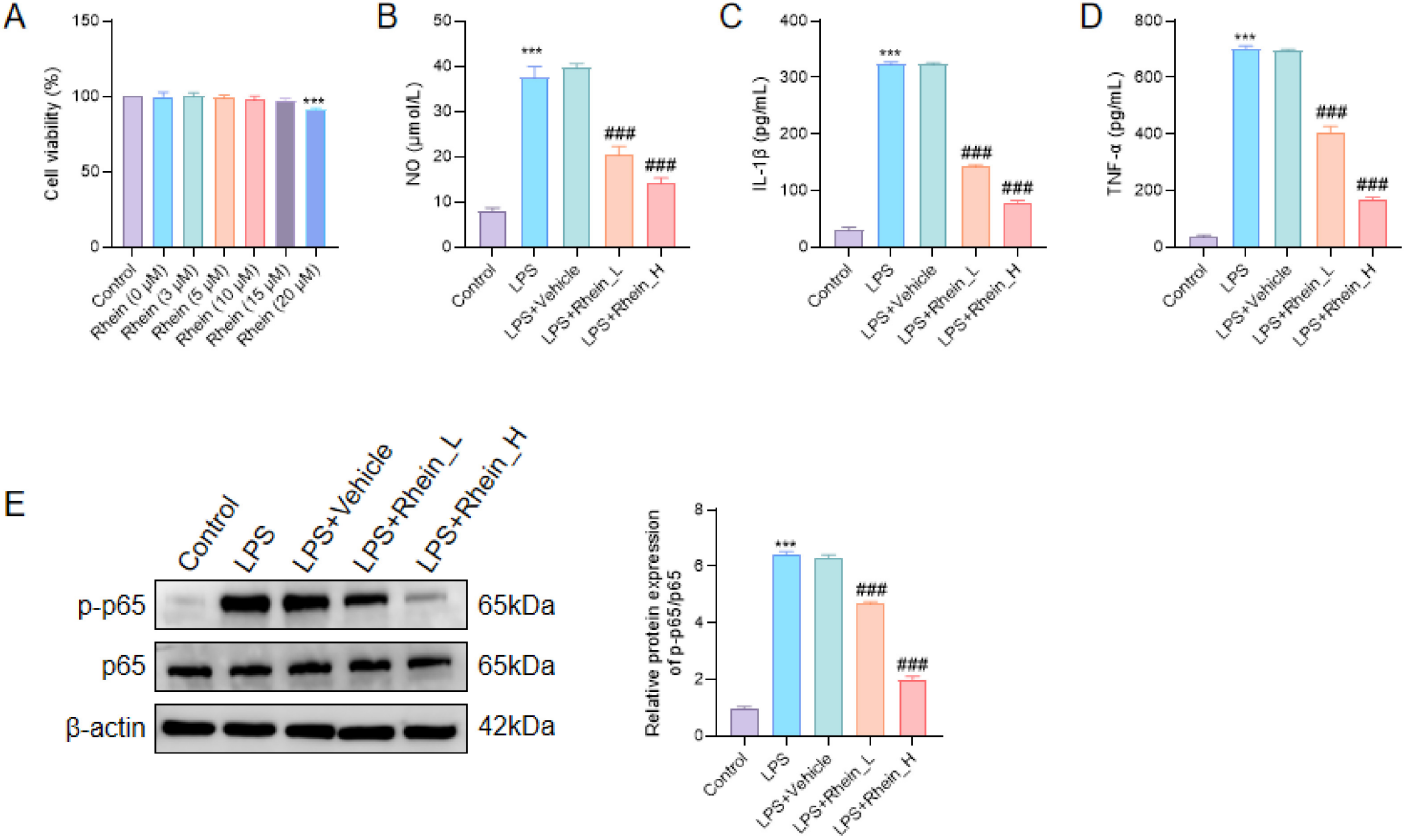
Effects of Rhein on BV2 microglial cells. **(A)** Cell viability of BV2 cells treated with different concentrations of Rhein, measured by CCK-8 assay. **(B)** NO levels in culture supernatants of Control, LPS, LPS + Vehicle, LPS + Rhein_L (3 μM), and LPS + Rhein_H (15 μM) groups. **(C,D)** Levels of IL-1β and TNF-α in cell supernatants determined by ELISA. **(E)** Representative Western blot bands and quantitative analysis of p-p65/p65 expression in BV2 cells across treatment groups. The data are presented as mean ± SD, *n* = 3. ***p < 0.001 vs. Control group; ###*p* < 0.001 vs. LPS group.

Cell viability assay indicated that Rhein treatment at concentrations ranging from 3 to 15 μM did not significantly affect BV2 cell survival, whereas a decrease was observed at 20 μM (*p* < 0.001) (Figure 7A). In the LPS-induced neuroinflammation model, compared with the control group, the level of NO significantly increased, and Reine treatment could reduce the production of NO, and the effect at a high concentration was more obvious than that at a low concentration (95% CI: 19.11–27.38, *p* < 0.001) (Figure 7B). ELISA results showed that LPS stimulation increased the secretion of cytokines IL-1β and TNF-α, while Reine pretreatment significantly reduced the levels of IL-1β (95% CI: 234.5–253.5, *p* < 0.001) (Figure 7C) and TNF-α (95% CI: 503.2–560.5, *p* < 0.001) (Figure 7D). Western blot analysis showed that LPS stimulation upregulated the phosphorylation of p65, while Reine pretreatment reduced the ratio of p-p65/p65 in BV2 cells (95% CI: 4.233–4.646, *p* < 0.001) (Figure 7E).

## Discussion

In the present study, a network pharmacology-based approach was combined with molecular docking and experimental validation to investigate the anti-neuroinflammatory effects of *Cassia obtusifolia* in PD. Bioinformatics analyses identified 14 bioactive compounds and 114 potential therapeutic targets, with PPI network analysis highlighting 10 hub genes closely related to inflammatory regulation. Functional enrichment revealed that these targets were mainly involved in key signaling pathways including NF-κB, MAPK, IL-17, Toll-like receptor, and NLRP3 inflammasome. Molecular docking revealed that rhein exhibited a strong binding affinity to NF-κB p65, and subsequent *in vitro* experiments confirmed that rhein attenuated LPS-induced neuroinflammatory responses in BV2 microglial cells by reducing NO, IL-1β, and TNF-α secretion, as well as suppressing p65 phosphorylation.

Previous studies have shown that neuroinflammation, particularly microglial activation and NF-κB signaling, plays a central role in PD progression (Li et al., 2021; Li et al., 2024). In comparison, our study not only confirmed the involvement of NF-κB and other classical inflammatory pathways but also systematically identified multiple hub genes and interconnected pathways through network pharmacology. Moreover, by integrating molecular docking and cell-based validation, we demonstrated that rhein directly interacts with NF-κB p65 and decreases its phosphorylation in BV2 microglia (Qin et al., 2024), providing mechanistic evidence that complements and extends earlier findings. By contrast, unlike prior work focusing on isolated compounds or single pathways, our approach highlights the multi-target and multi-pathway features of *Cassia obtusifolia*, thereby offering a more comprehensive understanding of its anti-neuroinflammatory potential in PD.

The multi-component and multi-target nature of *Cassia obtusifolia* suggests that its anti-neuroinflammatory effects are not restricted to a single molecular pathway. Rhein, emodin, and chrysophanol may act synergistically to modulate several signaling cascades (Li et al., 2019; Zheng et al., 2021). Among these, NF-κB emerged as a central hub, consistent with its established role as a master regulator of microglial activation and cytokine production. Docking analyses supported the potential direct interaction between rhein and NF-κB p65, and *in vitro* results confirmed a reduction in p-p65 levels after rhein treatment. This finding provides a plausible mechanistic link between compound–protein binding and downstream functional effects. In addition, enrichment analysis also implicated the MAPK and NLRP3 inflammasome pathways, suggesting that *Cassia obtusifolia* may exert broader immunomodulatory actions that warrant further experimental exploration (Fu et al., 2025; Khan et al., 2025). Collectively, these findings suggest that *Cassia obtusifolia* may mitigate neuroinflammation through a coordinated regulation of multiple signaling nodes, thereby offering a pharmacological rationale for its traditional use.

In the context of existing preclinical evidence, our findings align with but also extend prior studies evaluating *Cassia obtusifolia* and its major anthraquinone constituents in neurodegenerative disease models. Ju et al. reported that *Cassia obtusifolia* seed extract improved dopaminergic neuron survival and behavioral performance in MPTP-treated mice, focusing primarily on phenotypic outcomes. Similarly, Qin et al. demonstrated that rhein attenuated microglial activation and motor deficits through MAPK/IκB signaling in a PD mouse model. These *in vivo* results support the anti-neuroinflammatory potential of both the herb and rhein; however, the underlying mechanisms were not comprehensively characterized. In comparison, our study provides targeted mechanistic insights by demonstrating that rhein directly interacts with NF-κB p65 and significantly suppresses pro-inflammatory signaling in microglia, while network pharmacology analysis reveals multi-pathway involvement extending beyond NF-κB to MAPK and the NLRP3 inflammasome. Thus, our integrative approach adds mechanistic depth to prior reports and highlights the broader polypharmacological potential of *Cassia obtusifolia*. However, it must be noted that the current evidence for Cassia obtusifolia and Rhein in PD remains predominantly preclinical, with a notable absence of clinical trials to date.

Despite these promising findings, the translational relevance of rhein requires careful appraisal. Pharmacokinetic studies indicate that rhein exhibits only limited blood–brain barrier permeability, though measurable brain concentrations have been reported in rodents after systemic administration. This suggests that its neuroprotective effects may arise from both direct central actions and peripheral immunomodulation. Safety considerations also warrant attention, as prolonged or high-dose exposure to anthraquinones has been linked to gastrointestinal irritation and hepatotoxicity. The concentrations used in our BV2 assays fall within the non-cytotoxic range, but comprehensive toxicology and dosing studies are essential to determine safe therapeutic windows for neurological applications. Moreover, formulation strategies—such as nanoparticle delivery or prodrug modification—may enhance brain bioavailability and reduce systemic exposure.

Future research should therefore expand along several critical directions. First, *in vivo* validation using established PD models (e.g., MPTP, rotenone, or α-synuclein PFF models) will be necessary to confirm that the anti-inflammatory effects observed in microglia translate to meaningful neuroprotection in physiological systems. Second, systematic pharmacokinetic and pharmacodynamic profiling is needed to characterize rhein’s absorption, distribution, metabolism, brain penetration, and optimal dosing regimen. Third, given that *Cassia obtusifolia* contains multiple bioactive anthraquinones—including emodin and chrysophanol—combination assays should be performed to evaluate potential synergistic or additive effects that may more faithfully represent the multi-component nature of the herbal extract. Finally, toxicology assessments and safety evaluations will be indispensable before moving toward clinical development.

Overall, the present study provides early mechanistic insights into how rhein and other components of *Cassia obtusifolia* may modulate neuroinflammation in PD. Nevertheless, these *in vitro* findings should be interpreted as preliminary and will require robust *in vivo* and translational studies to determine their therapeutic significance.

## Conclusion

This study reveals the potential multi-target mechanism by which *Cassia obtusifolia* may regulate neuroinflammation. Rien inhibits the activation of microglia by suppressing the NF-κB signaling pathway, demonstrating its potential in the treatment of Parkinson’s disease. These findings indicate that the components of *Cassia obtusifolia* may become promising candidate substances worthy of further exploration in the research of neurodegenerative diseases, providing preliminary evidence for the application of traditional herbal medicine in neurodegenerative diseases.

## Data Availability

The original contributions presented in this study are included in this article/supplementary material, further inquiries can be directed to the corresponding author.

## Footnotes

1
https://tcmsp-e.com/

2
https://pubchem.ncbi.nlm.nih.gov

3
http://www.swisstargetprediction.ch

4
https://www.uniprot.org

5
https://www.genecards.org

6
https://www.disgenet.org

7
https://omim.org

8
https://string-db.org

9
https://cytoscape.org

10
https://www.rcsb.org

11
http://vina.scripps.edu

12
https://pymol.org
